# Clinician Perspectives on Palliative Care for People with Hepatocellular Carcinoma: Facilitators of and Barriers to Referral

**DOI:** 10.3390/cancers15143617

**Published:** 2023-07-14

**Authors:** Christopher D. Woodrell, Christie N. Mulholland, Nathan E. Goldstein, Carole L. Hutchinson, Thomas D. Schiano, Lissi Hansen

**Affiliations:** 1 Brookdale Department of Geriatrics and Palliative Medicine, Icahn School of Medicine at Mount Sinai, New York, NY 10029, USA; 2 Geriatric Research, Education and Clinical Center, James J. Peters Veterans Affairs Medical Center, Bronx, NY 10468, USA; 3 Mailman School of Public Health, Columbia University, New York, NY 10032, USA; 4 Department of Medicine, Icahn School of Medicine at Mount Sinai, Recanati/Miller Transplantation Institute, Division of Liver Diseases, New York, NY 10029, USA; 5 School of Nursing, Oregon Health and Sciences University, Portland, OR 97239, USA

**Keywords:** hepatocellular carcinoma, palliative care, supportive oncology, qualitative research

## Abstract

**Simple Summary:**

Hepatocellular carcinoma (HCC) is the most common type of liver cancer. HCC is difficult to cure and rates are rising. Treatment advances over the last decade have led to people living longer with HCC. Palliative care services offer potential additional support, but they receive referrals infrequently compared with other cancers. We need a better understanding of when these teams do and do not make palliative care referrals. We interviewed HCC-treating clinicians and found a number of relevant factors that impact referral. They refer at times of transition, for help with certain symptoms, and for additional support. They hesitate to refer because of lack of availability or too many appointments for the patient, not knowing who is appropriate, presence of symptoms they can manage themselves, and worry that the patient will think they have lost hope. These findings can be addressed when developing future palliative care programs for people with HCC.

**Abstract:**

(1) Background: Little is known about facilitators of and barriers to palliative care referral for people with hepatocellular carcinoma (HCC). The objective of this study is to identify facilitators and barriers of palliative care referral described by HCC-treating clinicians. (2) Methods: Semi-structured interviews (*n* = 16) were conducted with HCC-treating clinicians at two centers, focusing on referral patterns, palliative care needs, and disease course. A code book was created, axial coding was used to code all interviews, and selective coding was used to identify facilitators and barriers of palliative care referral. (3) Results: Facilitators included helpfulness at times of transition; help with management of certain symptoms; provision of psychosocial support; and positive experiences with referral. Barriers included feasibility concerns; lack of information about palliative care and who is appropriate; lack of symptoms requiring outside referral; and concerns that palliative care conveys loss of hope. (4) Conclusions: Participants noted the helpfulness of palliative care at specific points in the disease trajectory and cited barriers related to feasibility, lack of need, lack of awareness, and loss of hope. The results show actionable issues that can be addressed in future research to leverage the benefits of and overcome the barriers to palliative care for people with HCC.

## 1. Introduction

Hepatocellular carcinoma (HCC) is the most common type of primary liver cancer and a feared complication of chronic liver disease [1]. HCC is the fourth leading cause of cancer-related mortality worldwide [2] and is difficult to cure. Despite screening recommendations, only about a third of cases are identified early enough to allow for curative therapies such as radiofrequency ablation, surgical resection, and liver transplantation. Age, limited organ availability, psychosocial factors, and co-morbid illness further preclude these treatments for many individuals, even with early diagnosis. There also exist significant sociodemographic and particularly racial disparities in the receipt of standard treatments [3]. The overall survival of people with HCC is increasing, and numerous treatments have been approved over the last few years for the treatment of HCC, including immuno- and combination therapies [4,5].

The benefits of early palliative care in the ambulatory setting have been described in numerous solid malignancy-based populations [6,7], and there has been an enormous increase in the prevalence of supportive oncology practices, which include palliative care specialists, across the U.S. over the last decade [8]. Furthermore, in a recent Guidance Statement from the American Association for the Study of Liver Diseases about HCC management, palliative care is an essential component of the multidisciplinary tumor board [9]. Yet, there is evidence that palliative care is not reaching HCC patients and their families until later in the course of illness, and sometimes not at all [10]. There may be several factors that inhibit palliative care referral in cases where it might be helpful to patients and families. For instance, hepatologists and transplant surgeons are instrumental in providing care to these patients, and survey data suggest that some may be hesitant to refer to palliative care for fear of giving the impression of giving up hope [11,12]. In addition, palliative care in the ambulatory setting is not uniformly available [13], particularly in these practice settings. Finally, prognostic uncertainty may further inhibit timely referral to palliative care [14].

**Figure 1:**
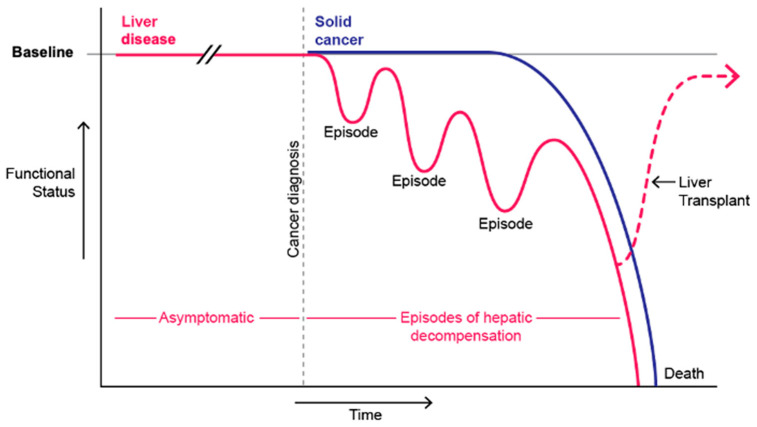
Functional status over time based on the disease trajectory models described by Murray et al. [21]; the figure depicts the trajectories of solid cancer (purple line) and end-stage liver disease (magenta line). HCC can feature both types of trajectories and may be punctuated by episodic decompensation, as is the case in the end-organ failure trajectory. Figure created by Jill K. Gregory, MFA, CMI.

We know that people living with HCC face symptom burden and an unpredictable disease course [15,16]. The need for symptom management, family caregiver support, communication, and psychosocial support may offer opportunities for palliative care to complement the care received from HCC specialists; however, identifying these time points may be challenging given the unpredictability of the course [17]. While models such as Hawley’s Bow Tie model [18] are useful to illustrate the increasing importance of palliative care relative to disease-directed therapy as a disease progresses, they do not necessarily reflect the variable course of HCC, particularly when superimposed with the disease course of cirrhosis (Figure 1). We also need a better understanding of which specific elements of palliative care are most helpful, and when, and of the factors that inhibit referral so as to tailor and optimize palliative care delivery for people facing HCC.

The objective of this study is to describe factors that promote (facilitators) or inhibit (barriers) palliative care referral from the perspectives of healthcare professionals across multiple specialties who provide care to people with HCC.

## 2. Materials and Methods

### 2.1. Study Design

This was a qualitative cross-sectional study that employed methods of constructivist grounded theory [19,20] and was designed to gather information about how and when clinicians refer to palliative care as well as their views on palliative care and the needs of their patients. Those data were used to selectively code and identify facilitators and barriers of palliative care referral for this clinician population. The Mount Sinai Program for the Protection of Human Subjects office approved the study as exempt human research, as defined by the Department of Health and Human Services (45 CFR 46.101[b]).

### 2.2. Participant Recruitment

The study team used purposive sampling to achieve a representative selection of individuals from the HCC-treating clinical realm. In order to achieve this, study participants were recruited from two academic medical centers with liver cancer treatment programs that include liver transplantation, located in New York and Oregon. Clinician participants included those working in the fields of nursing, medicine, and social work who routinely see patients with HCC and work in practice settings involved in the management and treatment of HCC (including medical, surgical, and radiation oncology, and palliative care practices).

### 2.3. Data Collection

The study team consisted of four physicians (CW, CM, TS, and NG), one nurse scientist (LH), and one anthropologist (CH). The team developed a data collection and analytic plan following a Constructivist Grounded Theory approach [19,22,23]. The semi-structured interview guide was developed with the disease trajectories shown in Figure 1 in mind, and focused on palliative care referral patterns, needs, disease course, and symptoms, but allowed space for participants to provide information unique to their interdisciplinary experiences caring for these patients. Specifically, the interview guide consisted of a series of open-ended questions, as well as prompts for additional questions asked when certain topics were mentioned.

Six pilot interviews (CW and CM) were supervised (CH) and conducted jointly to pilot test the interview guide and standardize the interviewing approach. This allowed the development of a parallel and consistent interviewing style among the interviewers. The interview guide was updated iteratively throughout the first six pilot interviews (which were not included in the study) to assure that the analytic motifs and objectives of the study could be captured in the most accurate and consistent manner. Thirty-minute (ranging 23–39 min, averaging 29 min) semi-structured study interviews (*n* = 17, one of which was excluded because the interview subject did not care for many HCC patients in any significant capacity) were conducted by at least one of two investigators and digitally recorded for accuracy.

All but one interview (which was conducted virtually for logistical reasons) were conducted in person in private conference rooms to allow for privacy, neutrality, and to assure the best environment for recording. Potential participants were scheduled for the interview and informed of who would conduct their interview. Each participant was given a sheet describing the study details, including the background information of the research purpose, study procedures, potential risks, anticipated benefits, and confidentiality assurances. The participants were told that the interview was completely voluntary and that they could refrain from answering any questions they did not want to answer and end the interview at any time. Digital recordings were transcribed by a HIPAA-compliant transcription service [24]. Transcripts were verified verbatim using the audio recordings.

### 2.4. Data Analysis

As noted above, Constructivist Grounded Theory, a research method that focuses on inductive analysis of the data gathered from participants, formed the basis for the study design and analytic plan. Additionally, while there are pre-existing descriptions of clinician-level factors that impact palliative care referral patterns in other clinical contexts (e.g., colorectal cancer, gynecology/oncology, and the emergency department [25,26,27]) that we considered in the construction of our interview guide, little was known about unique factors that impact referrals by HCC-treating clinicians. HCC is distinct from other types of solid cancer in terms of biology (frequent presence of concurrent cirrhosis), treatment modalities (systemic treatment is generally reserved for advanced disease), and a uniquely complex clinical landscape [9].

First, open coding was used to create a codebook using the first three study interviews, which were reviewed by three investigators (CLM, CDW, and CH). Second, axial coding [28] was then used to code all interviews, with quotations associated with relevant codes. The codebook and definitions were updated iteratively by consensus due to evolving understanding as the dataset grew. Eight categories of codes were identified and relevant quotations were associated with codes within these categories. Third, from these eight categories using selective coding, factors that affect whether or not palliative care referral occurs for HCC patients were identified from the existing codes. Throughout the process at least two investigators coded the data independently, but quotations from the transcripts were only placed under the different codes after consensus between the two investigators was reached through face-to-face discussion. Six of the sixteen interviews were coded by a third investigator to further ensure consistency. Thematic saturation [29], the point at which no new data emerged, was determined by agreement among all investigators, and occurred after the tenth interview. Sixteen interviews were conducted because study participants were identified as eligible and their interviews were included to diversify the different specialties and disciplines represented in the study.

## 3. Results

A total of 16 study interviews were included in the analysis. The interviews were conducted with treating clinicians from liver cancer programs in New York (*n* = 9) and Oregon (*n* = 7), and participants included physicians (*n* = 9), nurses (*n* = 6), and a social worker. The practice settings or specialties of the participants included hepatology (*n* = 6), liver surgery (*n* = 4), supportive oncology/palliative care (*n* = 2), medical oncology (*n* = 3), and radiation oncology (*n* = 1). Saturation was achieved at the tenth interview, at which point no new codes emerged.

Selective coding revealed eight categories that either promote (four) or inhibit (four) referral to palliative care for people with HCC. Types of factors that promoted or facilitated palliative care referral included helpfulness of palliative care at times of transition, need for help with symptom management, need for additional resources to manage psychosocial needs, and positive experiences with referrals leading to subsequent referrals. Types of barriers or inhibitory factors included feasibility concerns, lack of information about palliative care, lack of need for help with symptom management, and concerns about palliative care (e.g., referral conveying a loss of hope).

### 3.1. Facilitators of Palliative Care Referral

#### 3.1.1. Helpfulness at Times of Transition

While there may not always be a role for palliative care for all people throughout their disease course, participants cited times during the HCC trajectory where patients may have more palliative care needs. Specifically, multiple people identified times of transition as ideal times for palliative care referral, including loss of transplant eligibility, transition to systemic therapy, and discussions around transition to hospice. In reference to loss of transplant eligibility, one participant noted that after doing “this huge workup for transplant and then all of a sudden something else came up [such that] they are no longer a candidate, now what do we do?” Referral to medical oncology for systemic treatment was also cited as a transition point where palliative care could be helpful. At one center, referrals at the time of oncology referral are common: “when we [hepatology] refer to oncology, we also do a palliative care consult”. One palliative care provider who sees many HCC patients noted that the point at which someone is referred to medical oncology can be “like a tipping point for them. They may have lived with HCC for months or years…but now it’s different because they’re seeing an oncologist. Additionally, that is different because before they had cancer, but they did not necessarily see an oncologist”.

#### 3.1.2. Helpful for Management of Certain Symptoms

The presence of certain symptoms was also cited as a reason providers may consider a palliative care referral. These symptom-based facilitators include a need for the management of cancer-related pain, anxiety, and insomnia. One nurse in a liver surgery practice noted that “opioids [are often] a drug of choice…for short-term post-op pain, but the knowledge of tools available and comfort with treating [cancer] pain is limited in liver surgery”. One hepatologist similarly noted that they did not feel they are the ideal person to treat symptoms such as sleeping difficulties or anxiety, and that after trying a treatment modality and finding it did not work, would prefer to refer for help with the management of these symptoms.

#### 3.1.3. Provision of Psychosocial Support

The availability of additional psychosocial support was also discussed as a facilitator of palliative care referral. Numerous participants cited the need of this patient population and their family members for additional support and additional resources. For individuals who are no longer eligible for liver transplant, there may be less psychosocial support available without the interdisciplinary transplant team, as may be the case at some centers. One participant from a liver transplant program wondered “if [the patients] feel ‘nobody cares about me anymore, because…I am off the list;’ they’re still getting ongoing treatment, but it’s a shift and I wonder if in that shifting process it would be helpful to [offer palliative care]”.

#### 3.1.4. Positive Experiences with Referrals

Finally, participants noted that when palliative care services are available and once they do refer, witnessing the helpfulness of palliative care may promote subsequent palliative care referrals. One participant noted, “I think the only way to move palliative care upstream is…to have [HCC-treating clinicians] see the benefit themselves; they…have to have a [positive] clinical outcome for the patient”.

### 3.2. Barriers to Palliative Care Referral

#### 3.2.1. Feasibility Concerns

Participants cited concerns about the feasibility of involving palliative care for people with HCC, including the lack of consistent palliative care availability as well as adding an additional appointment to an already busy visit schedule for patients, who frequently see multiple specialists.

Multiple clinicians (both palliative care and HCC-treating) cited palliative care as a limited resource. One palliative care provider noted that the problem of availability may only become worse in the future, given “we’re a long way from having the…capacity in terms of clinician volume especially with…prevalence [of HCC] going up, and the patients who have it are having prolonged survival because we have effective treatments”.

In addition, one hepatologist participant noted that the treatment “of liver cancer has advanced so much that we [are] sending people from medical oncology to radiation oncology to interventional radiology…[there is] a lot of coordinating”. Having a “new provider who’s in a different location, an additional day coming back, is a lot for a patient”. Furthermore, if people are referred late in the course of HCC, getting to an additional appointment may be even more difficult, given they may become more debilitated quickly: “when [patients] feel sicker and sicker, [they] do not always show up for appointments”.

#### 3.2.2. Lack of Information

Non-palliative-care participants in the study cited lack of information or understanding about the services that palliative care provides as a barrier to referral, including not being clear on who and when to refer. One nurse from an HCC-treating practice noted “to be honest I do not know a lot about everything that palliative care does”. Because clear criteria for referral are lacking, it may not be clear which patients might benefit.

#### 3.2.3. Lack of Symptoms Requiring Outside Referral

Participants, particularly hepatologists, noted barriers related to symptom management, including first that the hepatologists or other specialists will manage any symptoms related to underlying liver disease and do not need palliative care’s help with those symptoms, and second that the symptoms that providers may need help managing, such as severe pain, are not common before someone’s disease becomes very advanced. A hepatologist noted that “after liver-directed treatment they can get ascites, sometimes encephalopathy…I am pretty comfortable dealing with those symptoms”. Multiple participants noted that “I’ve generally found pain to be…less of a significant issue” and “[patients] do not usually have pain early on unless they have a very big tumor that is causing stretching of the liver capsule”.

#### 3.2.4. Concerns about Palliative Care

A fourth type of barrier was concerns about palliative care, including seeing palliative care as being inconsistent with curative therapies, worry about patients perceiving loss of hope on the part of the treating team, and worry about introducing ambivalence about treatments to patients. One participant who was a nurse in a transplant program noted that “in order to get through the transplant process, you have to be really dedicated to doing a lot of invasive testing procedures…and I think there is maybe a fear of putting ambivalence in the patients’ minds”. Another noted that there may be some skepticism about palliative care: “palliative care means different things to different [people] (patients and also different providers), so I think…[palliative care for some] feels like giving up on someone”.

In addition, primary treatment teams may feel that their longitudinal involvement with some patients makes them better suited to complex discussions about care, rather than a referral to a new provider or team at the time of transition: “you rock the boat if…[you] introduce a new relationship and new practitioners and change all the interpersonal dynamics”.

## 4. Discussion

We conducted a qualitative study of HCC-treating clinicians’ views on referral to palliative care, needs related to palliative care, and their experience treating people with HCC. The data revealed both facilitators of and barriers to palliative care referral for people with HCC from the points of view of these clinicians. Participants identified specific areas of helpfulness of palliative care at certain points in the course of HCC, as well as barriers that exist both at the individual and systems levels. The different factors that participants noted to either promote or inhibit referral to palliative care for this population are noted in Table 1 and Table 2, respectively. In addition, we have noted ways that each factor might be promoted or addressed based on reports from populations of people facing other types of serious illness.

An important finding from this study was the significance of specific times of transition during the course of HCC. Participants cited specific milestones for people who do not undergo surgery or transplant, including time of removal from the liver transplant waiting list and initiation of systemic therapy. While many felt palliative care could be helpful at times of transition, they also cited the unpredictability of events such as liver decompensation or disease recurrence (Figure 1), thereby hindering the ability to provide timely referrals for additional support. One way to further support this phenomenon would be to identify specific patient and family needs at these milestones. There is indeed growing interest in the field of palliative oncology in the idea that palliative care can be delivered at specific disease-based milestones, with increased intensity and frequency of care delivery for those that are identified as having a higher level of need based on the use of a screening tool [30].

Participants identified two domains of palliative care as being helpful for the population of people with HCC: psychosocial support and, in some instances, symptom management. Psychosocial support was cited as something they view as an added benefit of referral to an interdisciplinary palliative care team. This came up in the context of support from social workers to help with care coordination, referral for services in the community, and family caregiver support, particularly for those who may have been removed from the transplant list and no longer have access to that interdisciplinary team. It is important to note, however, that not all palliative disciplines may be available within a given practice. Thus, while palliative care teams may provide the service of offering support, chaplaincy and social work are not always available, as demand has outpaced the growth of palliative care services in many settings [31]. Further investigation should identify opportunities to effectively and sustainably address psychosocial needs, which would not necessarily require palliative-care-specialized practitioners.

Interestingly, symptom management appeared in the dataset as both as a facilitator of and barrier to referral. Many palliative care studies in other cancer-based populations have found that the need for support in providing relief from symptoms forms a strong basis for early palliative care referral [6]. Participants cited symptoms that promote referral as including cancer-related pain, particularly when opioid prescriptions are needed, fatigue, insomnia, and psychological symptoms such as anxiety. However, those symptoms associated with portal hypertension and liver disease were noted by participants to be best managed by the liver specialists. These include ascites and encephalopathy, which are known to have the largest impact on caregiver quality of life [32]. There may be an opportunity to enhance collaboration given the liver disease specialist’s expertise in the management of liver disease symptoms and palliative care teams inter-professional models of communication. Future research directions may include enhanced primary palliative care (delivered by the HCC-treating teams) [33] and the use of patient-reported outcomes to identify those individuals who might have greater need and benefit from an additional team (e.g., a palliative care consultant team). Furthermore, there may be additional opportunity for enhanced communication between palliative care and HCC specialists through participation in multi-specialty case conferences.

Participants noted the limited availability of palliative care as a factor that can preclude referral, consistent with known shortages of palliative care-trained clinicians across health systems [33]. In addition, while palliative care has grown considerably in the last two decades, including in the outpatient setting and in cancer centers, the prevalence of outpatient services remains lower than inpatient services [13]. Our finding that referring patients to an additional appointment can be inhibitory to palliative care referral is not surprising given the complexity of the HCC treatment landscape, frequently necessitating visits to multiple sub-specialists [1]. People with HCC may also not be referred to palliative care because referring clinicians are not aware of services that might be provided or the services may be equated with hospice care [12]. Finally, a related finding was that there is the worry that the introduction of palliative care may introduce ambivalence and signify loss of hope to patients and families, which is seen in other reports of barriers to palliative care referral for people with serious illness [34]. Public perception of palliative care is a topic of important investigation, and future work should incorporate engagement with community and patient advocate organizations to learn more about how perceptions may serve as either barriers or facilitators of referral by clinicians.

The barriers identified by the participants of the study can be addressed to improve the consistency of palliative care referrals for people facing HCC. To address concerns about the complex logistics of attending yet another visit, models such as co-located or embedded palliative care practices [35] could be expanded to surgical and hepatology practices to enhance coordination and minimize inconvenience for patients and families. The advent of telehealth and rapid expansion during the COVID-19 pandemic [36] may also present an opportunity to maximize availability of palliative care for people facing HCC, especially for those who cannot easily travel to their treatment center. Finally, primary palliative care training for liver-disease-treating clinicians has garnered significant attention in recent years [37]. The implementation of clinician education programs could enhance the delivery of primary palliative care to people with HCC. Reservations about palliative care might be addressed through cross-specialty, bi-directional education and collaboration, as well as the normalization and increased availability of upstream supportive services.

This study has several strengths. First, while there is an existing body of literature on facilitators and barriers to palliative care referral among oncologists [38] and among hepatologists [11,12], this is the first study that examines clinician-level barriers and facilitators of palliative care referral in the context of HCC. Second, the study population represents diverse professional backgrounds and areas of specialty practice relevant to HCC patients. This study also has limitations. While the inclusion of participants from two centers allowed for more diverse geographical and institutional perspectives than a single-center study, there may be some differences in the experience of clinical providers at other centers. The findings are not generalizable to all practice settings, particularly those without availability of or resources to support specialty palliative care teams. Future implementation should focus on ensuring equitable access to palliative care for those that need it. Second, social desirability is a consideration given the use of semi-structured interviews for data collection. However, two investigators were able to conduct semi-structured interviews; in the case that a participant had a close professional relationship with the potential interviewer, the other investigator could conduct the interview alone. A third limitation is that the list identified from the interviews with HCC-treating clinicians is certainly not exhaustive. More research into both patient-level and systemic factors, paying particular attention to implementation frameworks, is needed to expand upon these findings.

## 5. Conclusions

We have identified heterogeneity in provider understanding and palliative care feasibility and availability, and have found that various time-points in the disease course for potential referral and types of palliative care services can be helpful for people living with HCC. As one participant said, “palliative care means different things to different people”. Our findings highlight the need for the development of supportive and palliative care interventions that are tailored to the individual needs and preferences—in terms of content, mode of delivery, and visit frequency—of people facing HCC, so that palliative care might better help treatment teams deliver care that is aligned with patient needs and preferences as they navigate living with HCC.

## Figures and Tables

**Table 1:** Descriptions of factors that promote palliative care referral for people with HCC from the qualitative dataset and ways to further investigate or develop these factors.

Factors That Promote Palliative Care Referral—Facilitators	Examples	Actions to Address Said Factor
Helpfulness at times of transition	Palliative care referral may occur in the setting of transplant list removal or initiation of systemic therapy; participants emphasized prognostic uncertainty	Evaluation and testing of automatic palliative care assessment at transition points Development of tailored communication interventions (e.g., frameworks to express prognostic uncertainty)
Help with symptom management	Palliative care can help manage cancer-related pain requiring opioids, insomnia, and anxiety	Use of patient-reported outcome measures to trigger palliative care referral
Provision of additional psychosocial support	Provision of additional support particularly in the setting of removal from transplant list	Proactive identification of people in need of additional psychosocial support, coordination with existing social work, and chaplaincy services
Positive experiences	Witnessing helpfulness of palliative care in practice	Development of inter-departmental feedback mechanisms for quality improvement

**Table 2:** Descriptions of factors that inhibit palliative care referral for people with HCC from the qualitative dataset and ways to further investigate or develop these factors.

Factors That Inhibit Palliative Care Referral—Barriers	Examples	Actions to Address Said Factor
Feasibility concerns	Lack of palliative care availability Adding additional visit to busy patient schedule	Development of co-located or embedded palliative care models; use of telehealth
Lack of information	Unclear referral criteria Not sure what services are offered by palliative care	Development of referral criteria and dissemination to HCC-treating clinicians
Lack of symptoms that require referral	Many symptoms managed primarily by liver specialists Pain not very prevalent until very advanced disease	Development of primary palliative care interventions, patient and family caregiver education
Concerns about palliative care	Worry about patient perception of ambivalence, doubt, and lost hope Worry about introducing new team	Broad messaging about the nature and benefits of early palliative care; normalize early identification of surrogate decision makers; consideration of language used to describe palliative services

## Data Availability

The data in this study are available upon request to the corresponding author.
